# Correlation Between Implant Stability Quotient and Percussion Sound Frequency

**DOI:** 10.1002/cre2.917

**Published:** 2024-07-07

**Authors:** Wansiri Peeraprasompong, Weerapan Aunmeungtong, Pathawee Khongkhunthian

**Affiliations:** ^1^ Center of Excellence for Dental Implantology, Faculty of Dentistry Chiang Mai University Chiang Mai Thailand

**Keywords:** bone density, implant stability, ISQ, percussion sound frequency, primary stability

## Abstract

**Objectives:**

To determine the correlation between the primary implant stability quotient and the implant percussion sound frequency.

**Materials and Methods:**

A total of 14 pigs' ribs were scanned using a dental cone beam computed tomography (CBCT) scanner to classify the bone specimens into three distinct bone density Hounsfield units (HU) value categories: D1 bone: >1250 HU; D2: 850–1250 HU; D3: <850 HU. Then, 96 implants were inserted: 32 implants in D1 bone, 32 implants in D2 bone, and 32 implants in D3 bone. The primary implant stability quotient (ISQ) was analyzed, and percussion sound was recorded using a wireless microphone connected and analyzed with frequency analysis software.

**Results:**

Statistically significant positive correlations were found between the primary ISQ and the bone density HU value (*r* = 0.719; *p* < 0.001), and statistically significant positive correlations between the primary ISQ and the percussion sound frequency (*r* = 0.606; *p* < 0.001). Furthermore, significant differences in primary ISQ values and percussion sound frequency were found between D1 and D2 bone, as well as between D1 and D3 bone. However, no significant differences were found in primary ISQ values and percussion sound frequency between D2 and D3 bone.

**Conclusion:**

The primary ISQ value and the percussion sound frequency are positively correlated.

## 1 Introduction

Implant stability, an indirect indicator of osseointegration, is a dental implant's functional mobility measurement that is critical to the long‐term success of dental implant treatment (Choi et al. 2011). Primary stability, also known as mechanical stability, and secondary stability, also known as biological stability, are two types of implant stability. The absence of clinical implant mobility as a result of an implant's interaction with the surrounding bone in contact is defined as primary stability, which is determined by various parameters, including local bone quality and quantity, implant geometry, and implant insertion technique (Meredith 1998). Secondary stability is the tissue reaction and bone remodeling process that occurs because of a biocompatible implant, causing osseointegration (Sennerby et al. 1991).

Measuring implant stability could support the surgeon in developing an optimal treatment plan, assist with decisions on implant load timing, improve communication between the surgeons, and improve case documentation. Implant stability has been assessed using various classified approaches: invasive/destructive or noninvasive/nondestructive. Invasive or destructive approaches included histologic or histomorphometry analysis, the gold standard for assessing osseointegration (Bosshardt, Chappuis, and Buser 2017), tensional test which was modified by Bränemark (Brånemark et al. 1998), push‐out or pull‐out test, and removal torque analysis, which was developed by Johansson and Albrektsson (Johansson and Albrektsson 1987; Roberts et al. 1984). However, due to ethical considerations associated with invasiveness and various limitations, invasive procedures are usually exclusively employed in preclinical settings and may be useful as research methods.

Invasive procedures cannot be employed in clinical settings. To overcome these limitations, in clinical practice, currently, a noninvasive technique is frequently used to assess implant stability. Noninvasive or nondestructive approaches include surgeon's assessment, which is based on the surgeon's perception, radiographic examination, cutting resistance/insertional torque assessment, modal evaluation, periotest, resonance frequency analysis (RFA), and percussion test.

Although various methods to evaluate implant stability have been proposed, a simple, predictable, noninvasive approach for measuring implant stability and osseointegration is highly desirable. Resonance frequency analysis, introduced by Meredith in 1998 (Meredith, Alleyne, and Cawley 1996), is a noninvasive method most commonly used to measure implant stability using vibration and a structural analysis concept (Aparicio, Lang, and Rangert 2006). Historically, the RFA method used a small L‐shaped transducer connected to the implant or abutment. The transducer consists of two ceramic pieces, including one that vibrates in response to a signal and one that functions as a receptor. The transducer is inserted straight into the implant fixture and vibrates at a continuous input and amplitude, starting low and then increasing in pitch until the implant resonates. More stable bone–implant contact is indicated by higher frequency resonance.

While several generations of RFA have been introduced, the most recent version of RFA employs magnetic technology through a transducer consisting of a magnetic peg attached to the implant or abutment. When the magnetic resonance frequency probe activates the peg, it vibrates and produces an electric volt that is recorded by the magnetic resonance frequency analyzer. At present, Osstell ISQ (Osstell AB, Gothenburg, Sweden), which is a magnetic resonance frequency analysis device that uses a SmartPeg transducer, is commonly used because of its simplicity of use and repeatability, as well as its accuracy and reliability (Brouwers et al. 2021; Bural, Dayan, and Geçkili 2020). When evaluating implant stability, the related SmartPeg transducer and Osstell ISQ detect the frequency of vibrations and convert it into an implant ISQ value.

The implant ISQ value is commonly used in clinical practice for case documentation and surgeon communication. In addition, the implant ISQ value is an indirect indicator for estimating the time interval for implant prosthetic loading and a prognostic indicator of implant failure. Impaired primary stability jeopardizes the osseointegration process (Roos, Sennerby, and Albrektsson 1997). The implant ISQ value ranges from 0 (lowest implant stability) to 100 (highest implant stability). According to various study results, three categories of primary stability at the surgical visit were established. On the ISQ scale, ISQ > 70 indicates high stability, ISQ 60–69 indicates moderate stability, and ISQ < 60 indicates low stability (Baltayan et al. 2016; Hicklin et al. 2016; Pagliani et al. 2013; Trisi et al. 2010).

In addition to the Osstell ISQ, a common dental procedure to evaluate implant stability is the percussion test, which involves tapping the implant with the back end of a metal mouth mirror to generate a distinctive sound wherein the sound generated when a metallic device is tapped can be used for the clinical evaluation of osseointegration. A resounding “crystal” sound suggests efficient osseointegration, whereas a “dull” sound signifies insufficient osseointegration (Rizzo 2020). However, the diagnosis of an integrated implant using percussion sound is subjective and based on the surgeon's assessment of the sound quality.

Although the percussion test is a simple diagnostic method, there are currently no studies demonstrating its reliability or association with implant stability, and the correlation and reliability of implant ISQ values and percussion tests are still being discussed. The purpose of this study was to examine whether there is any correlation between the primary ISQ value and the frequency of percussive sound.

## 2 Materials and Methods

### 2.1 Sample Size Calculation

The power calculation of this study was carried out to determine the sample size, which was calculated based on a pilot study in which 10 implants were inserted into two bone specimens with a probability of type I error of 0.05 and a probability of type II error of 0.20. A minimum of four implants per group were required. To prevent study error, this study assigned 32 implants to each group.

### 2.2 Study Design

- Bone specimens' preparation
  The study used bone specimens from pig ribs, which were selected and collected at a butcher's shop. The bone specimens were prepared as the soft tissues were removed and the bone was exposed. Each rib was scanned using a CBCT scanner (DentiiScan 2.0, NECTEC NSTDA, Pathum Thani, Thailand). On the cross‐sectional image, the position of each implant was outlined with at least 10 mm between each implant. Each selected site required:

1   Bone height ≥ 13 mm.
2   Bone thickness ≥ 7 mm

**Figure 1:**
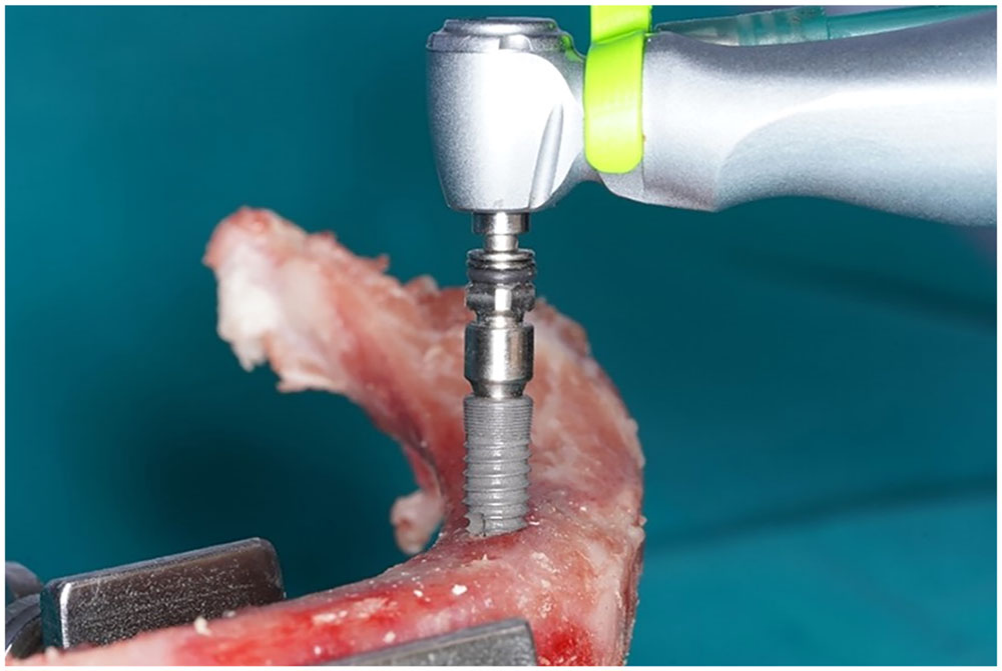
Implant insertion.

- Bone density evaluation
  At each implant site, the bone density HU value was measured to classify the bone specimens into three distinct bone density HU value categories (D1 bone: >1250 HU; D2: 850–250 HU; and D3–D4: <850 HU). The bone density HU value was evaluated three times using the annotations option of Planmeca Romexis Imaging Software (Planmeca Co. Ltd., Helsinki, Finland), the mean value was calculated, and each implant site was categorized into three categories based on bone density.
- Surgical procedures
  Each bone specimen was fixed to a bench vice to prevent micromovements during surgery. The implant sites were initially marked with a 1.8‐mm diameter round bur, then 2.0‐mm diameter pilot drills, then 2.8‐, 3.4‐, 3.8‐, and 4.6‐mm diameter twist drills 12 mm in depth. The implant fixtures PW Plus implants (PW Plus Co., Ltd., Nakorn Pathom, Thailand) with a diameter of 5.0 mm and length of 12 mm were then inserted with a handpiece adaptor at the bone level using 40 Ncm of insertion torque (Figure 1).

### 2.3 Outcome Variables and Measurements

**Figure 2:**
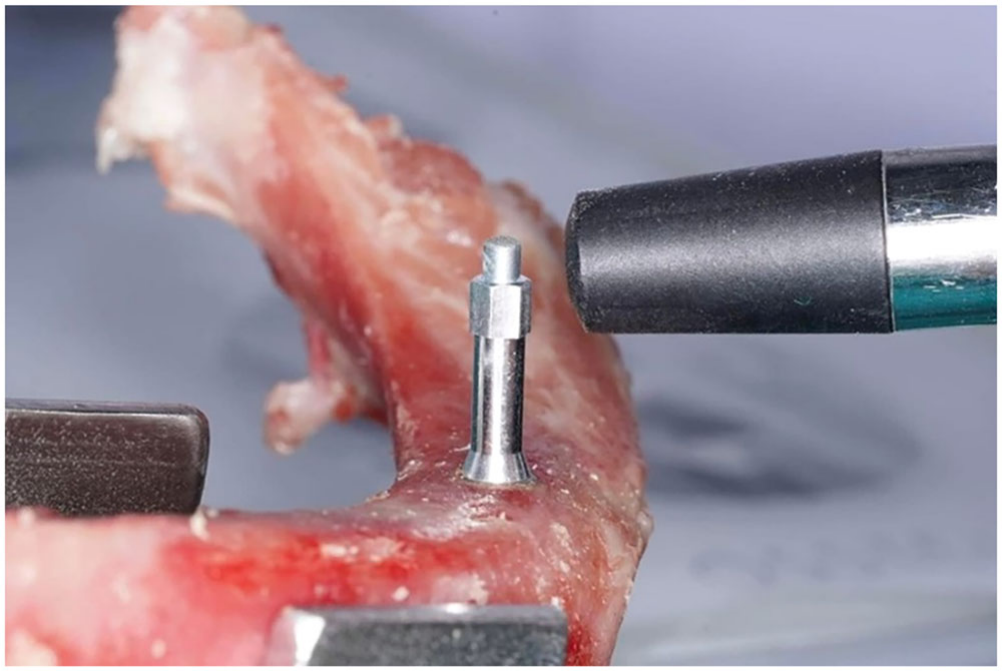
Primary ISQ measurement.

**Figure 3:**
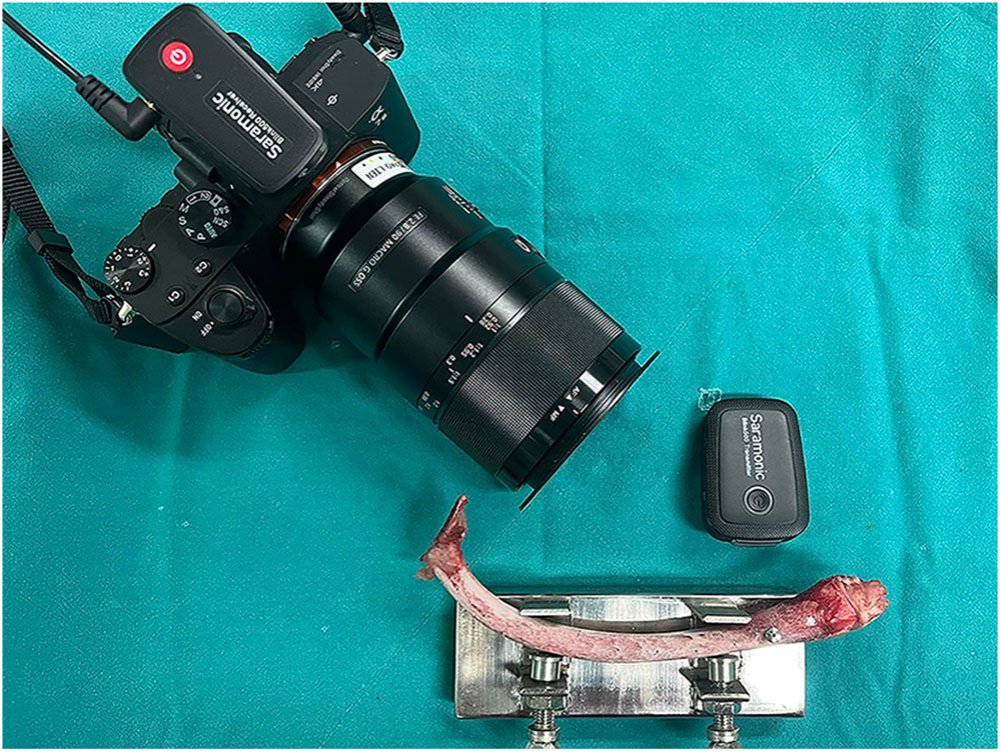
Percussion sound recording.

**Figure 4:**
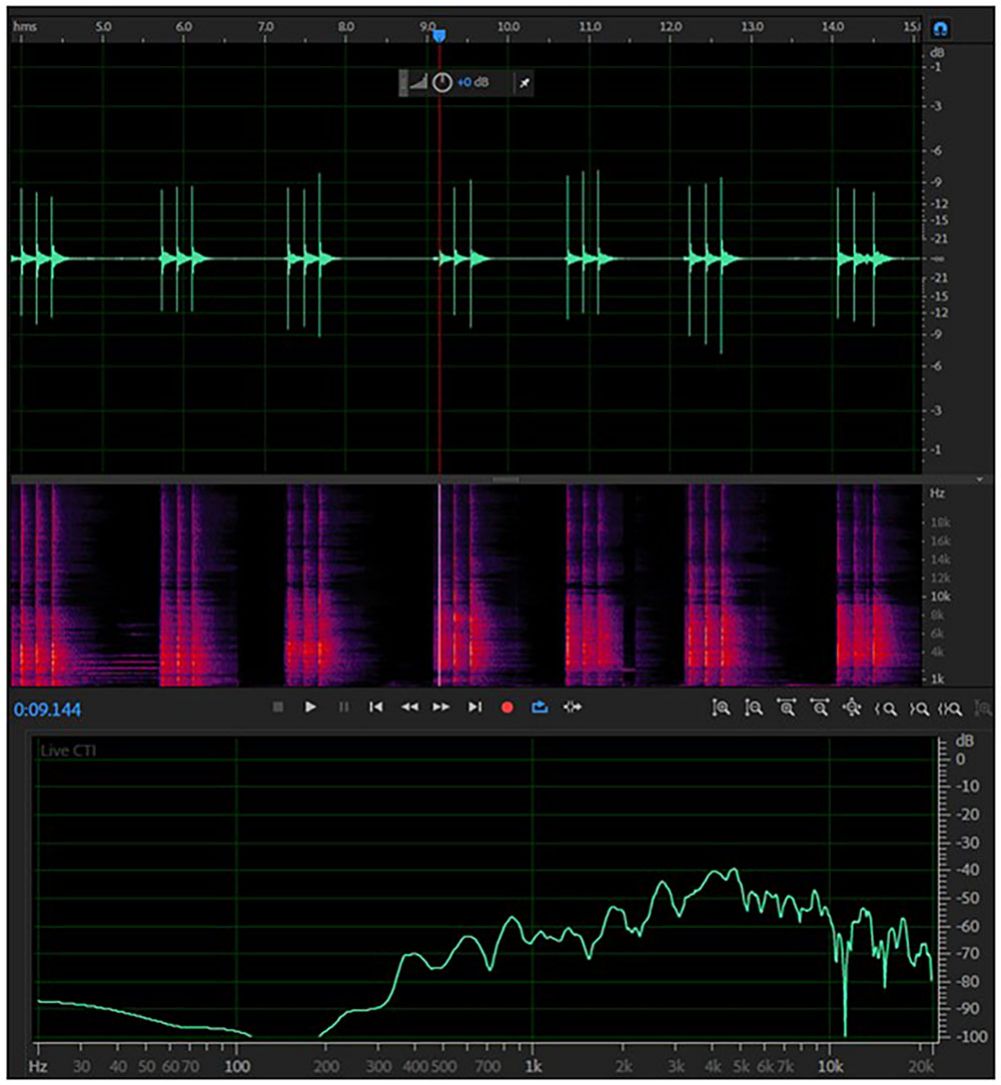
Sound frequency analysis software.

- Primary implant stability
  Following the insertion of implant fixtures into a bone specimen, the Smartpeg type 47 transducer was fastened securely to the implant fixture. An Osstell ISQ (Osstell AB, Gothenburg, Sweden) was used to measure the primary implant stability (Figure 2). Primary ISQ values were recorded in four directions transverse to the long axis of the bone specimen. Each direction was measured twice, the highest value was recorded, and the mean value was calculated for statistical analysis.
- Implant percussion sound frequency
  The implant percussion test was conducted by a single examiner in a quiet, enclosed room to ensure that no external sounds or noises interfered with the results. A percussion sound was recorded by firmly attaching the healing cap with a diameter of 5.8 mm and a height of 3.5 mm to the implant fixture using 15 Ncm of torque, following the manufacturer's instructions, and tapping the healing cap three times in a direction parallel to the implant's long axis using the back end of a metal mouth mirror. The percussion sounds were recorded using a wireless microphone: Saramonic Blink500 B2 (Saramonic, Shenzhen, China) which was placed at a distance of 5 cm from the implant (Figure 3). The sound frequency was analyzed using Adobe Audition CC 2018 V11.0.0.199 frequency analysis software with the noise reduction function to eliminate interfering noise from the sound (Figure 4), and the mean percussion sound frequency was calculated for statistical analysis.

### 2.4 Statistical Analysis

SPSS statistics were used to evaluate the data with a 5% level for significance. The reliability of intra‐raters to percuss implants was assessed to improve the accuracy and precision of the study. For each bone type, the mean and standard deviation of the primary ISQ values, as well as the frequency of the percussion sound, were calculated. The difference between the primary ISQ value and the percussion sound frequency in each bone type was determined using analysis of variance (ANOVA). The Pearson correlation test was used to determine the correlation between primary ISQ values and percussion sound frequency.

## 3 Results

### 3.1 Intra‐Rater Reliability (ICC)

The intra‐rater reliability was 0.971, demonstrating a high degree of agreement among repeated administrations of a percussion test performed by a single examiner.

A total of 96 implants were inserted in 14 pigs, ribs: 32 implants were inserted in D1 bone, 32 implants in D2 bone, and 32 implants in D3 bone (no D4 bone was found in the pig's rib).

### 3.2 Bone Density HU Value

The bone density HU value ranged from 590.24 to 1621.66. The mean bone density HU value was 1028.44 ± 262.98. In the D1, D2, and D3 bones, the mean density HU value was 1348.78 ± 84.08, 993.67 ± 98.70, and 742.86 ± 61.29, respectively (Table 1).

**Table 1:** Bone density HU value.

Group	Mean bone density HU value ± SD	Minimum	Maximum
D1 bone (*N* = 32)	1348.78 ± 84.08	1254.61	1621.66
D2 bone (*N* = 32)	993.67 ± 98.70	865.12	1208.04
D3 bone (*N* = 32)	742.86 ± 61.29	590.24	812.38
In total (*N* = 96)	1028.44 ± 262.98	590.24	1621.66

### 3.3 Primary Implant Stability

**Figure 5:**
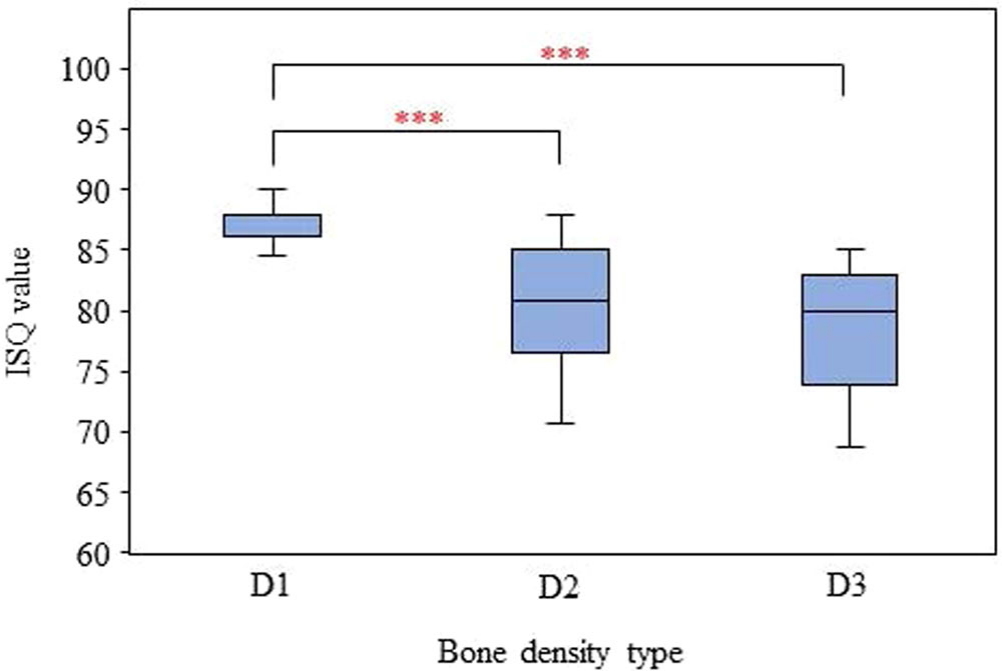
Comparison of the primary ISQ value between the different types of bones. Significant differences in the primary ISQ value between D1 and D2 and between D1 and D3 bone (*p* < 0.001).

**Figure 6:**
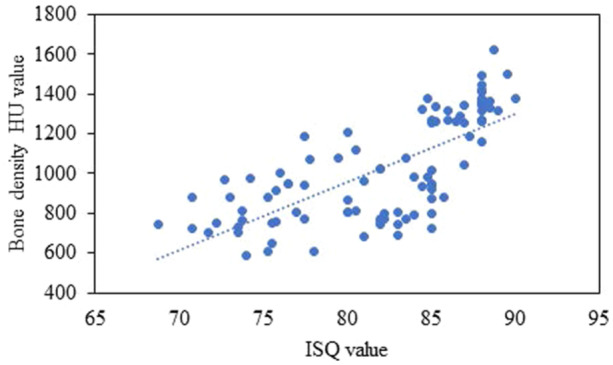
Scatterplot for the relation between primary ISQ value and bone density HU value.

The primary ISQ value ranged from 68.75 to 90.00. The mean primary ISQ was 82.07 ± 5.50. In the D1, D2, and D3 bones, the mean primary ISQ was 87.35 ± 1.44, 80.57 ± 4.79, and 78.28 ± 4.66, respectively (Table 2). The one‐way ANOVA results revealed significant differences in the primary ISQ value between D1 and D2 and between D1 and D3 bone (*p* < 0.001) (Figure 5). No significant differences were found between the primary ISQ values in D2 and D3 bone (*p* > 0.05) (Table 2). The Pearson correlation revealed a statistically significant correlation between the primary ISQ value and the bone density HU value (*r* = 0.719; *p* < 0.001). In other words, implants in areas with higher bone density tended to have a higher primary ISQ value than implants in areas with lower bone density (Figure 6).

**Table 2:** Primary ISQ value and bone density type.

Group	Mean primary ISQ value ± SD	Minimum	Maximum	Post hoc Dunnett T3 Test of mean primary ISQ value (*p* value)		
				D1 bone	D2 bone	D3 bone
D1 bone (*N* = 32)	87.35 ± 1.44	84.50	90.00			
D2 bone (*N* = 32)	80.57 ± 4.79	70.75	88.00	<0.001^a^		
D3 bone (*N* = 32)	78.28 ± 4.66	68.75	85.00	<0.001^a^	0.161	
In total (*N* = 96)	82.07 ± 5.50	68.75	90.00			

a The mean difference is significant at the 0.05 level.

### 3.4 Percussion Sound Frequency

**Figure 7:**
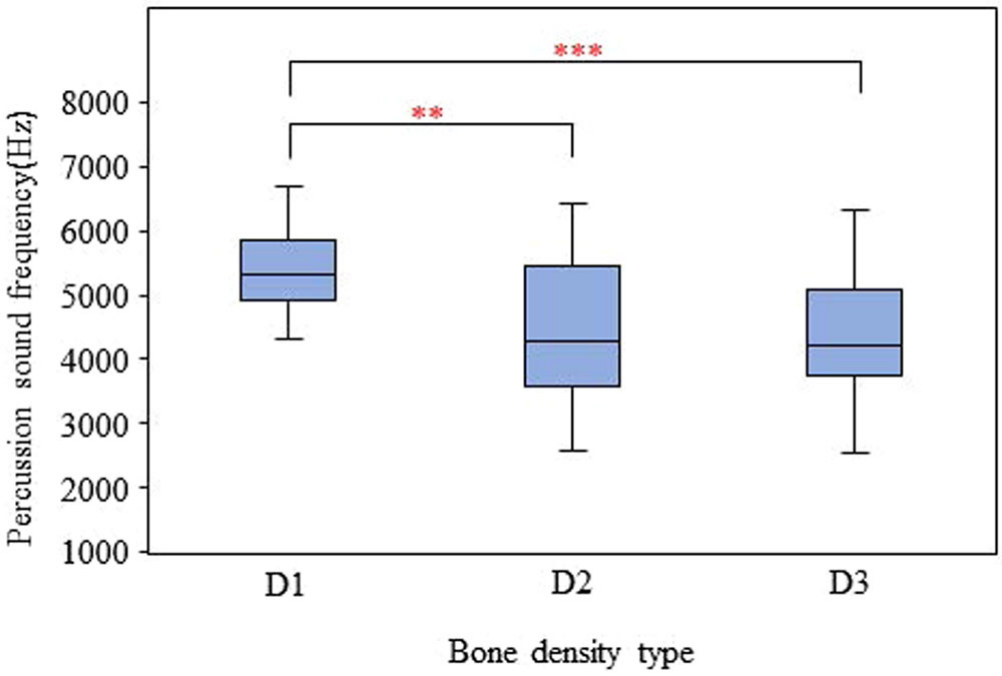
Comparison of the percussion sound frequency between the different types of bones. Significant differences in the percussion sound frequency between D1 and D2 (*p* = 0.001) and between D1 and D3 bone (*p* < 0.001).

The implant percussion sound frequency ranged from 2546.88 to 6703.13 Hz with a mean percussion sound frequency of 4759.40 ± 992.70 Hz. In the D1, D2, and D3 bones, the mean percussion sound frequency was 5377.24 ± 608.11, 4531.35 ± 1057.77, and 4369.62 ± 962.64 Hz, respectively (Table 3). The results of one‐way ANOVA revealed that there were significant differences in the percussion sound frequency between D1 and D2 (*p* = 0.001) and between D1 and D3 bone (*p* < 0.001) (Figure 7). No significant differences were found between the percussion sound frequency in D2 and D3 bone (*p* > 0.05) (Table 3).

**Table 3:** Percussion sound frequency and bone density type.

Group	Mean percussion sound frequency (Hz) ± SD	Minimum (Hz)	Maximum (Hz)	Post hoc Dunnett T3 Test of mean percussion sound frequency (P‐value)		
				D1 bone	D2 bone	D3 bone
D1 bone (*N* = 32)	5377.24 ± 608.11	4312.50	6703.13			
D2 bone (*N* = 32)	4531.35 ± 1057.77	2578.13	6437.50	0.001^a^		
D3 bone (*N* = 32)	4369.62 ± 962.64	2546.88	6312.50	<0.001^a^	0.891	
In total (*N* = 96)	4759.40 ± 992.70	2546.88	6703.13			

a The mean difference is significant at the 0.05 level.

### 3.5 Correlation Between the Bone Density HU Value and the Implant Percussion Sound Frequency

According to the study results, Pearson correlation analysis showed statistically significant positive correlations between the bone density HU value and the percussion sound frequency (*r* = 0.475; *p* < 0.001). This implies that implants inserted in locations with higher bone density tend to produce a higher percussion sound frequency than implants inserted in locations with lower bone density.

### 3.6 Correlation Between the Primary ISQ Value and the Implant Percussion Sound Frequency

**Figure 8:**
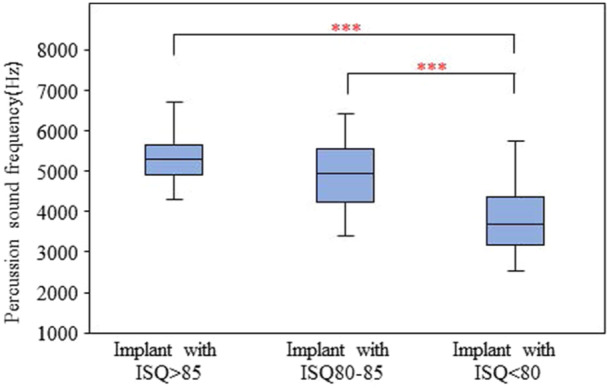
Comparison of the percussion sound frequency and range of the primary ISQ value. Significant differences in the percussion sound frequency between implant with primary ISQ > 85 and implant with primary ISQ < 80 (*p* < 0.001) and between implant with primary ISQ 80–85 and implant with primary ISQ < 80 (*p* < 0.001).

According to the study results, three groups based on primary ISQ values were classified. Implants with primary ISQ > 85 had an implant percussion sound frequency range of 3421.88–6703.13 Hz with a mean of 5272.26 ± 725.94, implants with primary ISQ 80–85 had an implant percussion sound frequency range of 3406.25–6437.50 Hz with a mean of 5012.04 ± 814.57, and implants with primary ISQ < 80 had an implant percussion sound frequency range of 2546.88–6140.63 Hz with a mean of 3888.58 ± 887.98 (Table 4). The results of one‐way ANOVA revealed that there were significant differences in the percussion sound frequency between implant with primary ISQ > 85 and implant with primary ISQ < 80 (*p* < 0.001) and between implant with primary ISQ 80–85 and implant with primary ISQ < 80 (*p* < 0.001) (Figure 8). No significant differences were found between the percussion sound frequency in implant with primary ISQ > 85 and implant with primary ISQ 80–85 (*p* > 0.05) (Table 4).

**Table 4:** Percussion sound frequency and range of primary ISQ value.

Group	Mean percussion sound frequency (Hz) ± SD	Minimum (Hz)	Maximum (Hz)	Post hoc Dunnett T3 Test of mean percussion sound frequency (*p* value)		
				ISQ > 85	ISQ 80–85	ISQ < 80
ISQ > 85 (*N* = 32)	5272.26 ± 725.94	3421.88	6703.13			
ISQ 80‐85 (*N* = 35)	5012.04 ± 814.57	3406.25	6437.50	0.428		
ISQ < 80 (*N* = 29)	3888.58 ± 887.98	2546.88	6140.63	<0.001^a^	<0.001^a^	
In total (*N* = 96)	4759.40 ± 992.70	2546.88	6703.13			

a The mean difference is significant at the 0.05 level.

**Figure 9:**
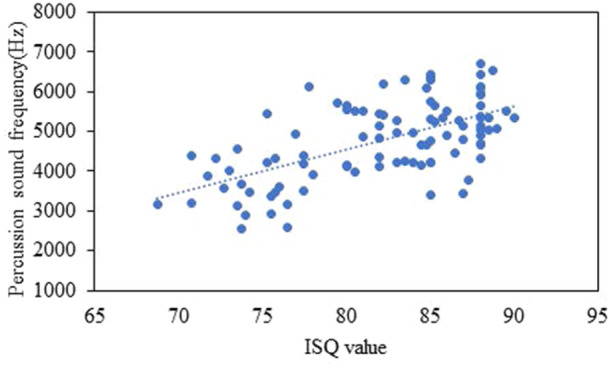
Scatterplot for the relation between primary ISQ value and percussion sound frequency.

The Pearson correlation revealed statistically significant positive correlations between the primary ISQ and the percussion sound frequency (*r* = 0.606; *p* < 0.001). In other words, higher implant percussion sound frequencies tend to have a higher primary ISQ value than lower implant percussion sound frequencies (Figure 9).

## 4 Discussion

Implant stability is one of the most important factors for successful dental implant treatment. According to previous studies, bone density has had an effect on implant stability where implants inserted in locations with high bone density demonstrated higher stability and success rates than those inserted in locations with poor bone density (Jemt and Lekholm 1995; Monje, Suarez, and Garaicoa 2014). However, in another study, bone quality had no effect on primary implant stability (Alsabeeha et al. 2010). Regarding the aspect of the value of ISQ and bone density type, there are many controversial views that may result from the different bone types' classifications, some of which are very rough and subjective. Consequently, this study classifies bone types using Hounsfield units assessed by CBCT scanner according to Misch's classification (Misch 1999), which provides a clear‐cut quantitative, reproducible basis for classification, and a bone density HU assessment using CBCT is an efficient method for implant planning and significantly correlated with the implant stability parameter (Fuster‐Torres et al. 2011).

According to the similarity to human mandibles in terms of bone homogeneity and cortical bone thickness (Friberg et al. 1995; Kim et al. 2010; Szalma et al. 2019), pig ribs were chosen for this in vitro study. A total of 14 pig ribs were scanned using a CBCT scanner, and only bone density types D1, D2, and D3 were detected as there was no bone type D4 detected. Therefore, only three bone density types were compared in this study.

In this study, bone density type D1 had a significantly higher primary ISQ value than bone density types D2 and D3, and significant positive correlations were found between the primary ISQ and the bone density HU value (*r* = 0.719; *p* < 0.001). This finding agrees with Turkyilmaz et al. (2007) who studied 142 implants and reported a significant correlation (*r* = 0.659; *p* < 0.001) between bone density HU value (751 ± 257) and ISQ values (70.5 ± 7) at implant placement. In a study of 23 implants in 10 subjects, Aksoy, Eratalay, and Tözüm 2009) also found a significant correlation (*r* = 0.807; *p* = 0.015) between HU values (554.87 ± 302.045) and ISQ values (72.78 ± 6.194). Merheb et al. (2010) found in a clinical study of 136 implants placed in 24 patients that a significant (*p* < 0.05) linear relationship was found between ISQ and HU values at implant placement and loading. Song, Jun, and Kwon (2009) reported a positive correlation (*p* < 0.025) between voxel values (grayscale) and ISQ values in a study of 61 implants in 20 patients.

In addition to the RFA device, which is used in implantology, the percussion test is a common screening procedure in dentistry. For normal healthy teeth, the periodontal ligament at the healthy bone–natural tooth interface attenuates the percussion energy generated by mastication. However, when a natural tooth must be replaced by an implant, there is no ligament involved, and the implant distributes percussion forces directly into the bone. Several studies show that percussion sounds can be used to diagnose problems in both natural teeth and implants (Heûdzelek and Hornowski 1998). Sound frequency analysis has been used to differentiate between healthy and periodontal teeth. The percussion sound in periodontal teeth was longer compared to healthy teeth because of periodontal tissue progress. (Taguchi et al. 1990). In a study of 79 teeth conducted by Karen et al. (Campbell et al. 2005), the frequency of percussion sounds was significantly higher in ankylosed teeth compared to non‐ankylosed teeth (*p* < 0.05).

Osseointegration of dental implants can be considered as a functional ankylosis (Schroeder et al. 1981). Failed osseointegration can be identified when implants exhibit mobility. One simple method to evaluate implant mobility is performing tapping tests using a blunt‐ended instrument. The Periotest is an electronic device developed specifically for this purpose. It uses a tapping head to percuss the implant and measures the time taken for the tapping head to make contact with the implant. The software in the instrument then translates the contact time to implant mobility (Oh et al. 2009). However, in implant dentistry, the Percussion test is one of the easiest tests to evaluate the level of osseointegration (Meredith 1998; Truhlar, Morris, and Ochi 2000) and is used by many clinicians (Adell 1985). A clinical evaluation of osseointegration is based on the sound produced by metallic percussion instruments. A “crystal” sound that rings clearly, which is a high‐frequency sound, means successful osseointegration, while a “dull” sound means osseointegration is not going well (Rizzo 2020). Although sound characteristics can indicate implant stability, this method is not a standard procedure because it is dependent on the clinician's experience, which is nonmeasurable. Consequently, this study converts the percussion sound into a frequency of sound that can be measured.

In this study, the difference in percussion sound frequency among bone density types was evaluated. Bone density type D1 had a significantly higher percussion sound frequency than bone density types D2 and D3, which corresponds to primary ISQ values that were significantly higher for bone density type D1 than for types D2 and D3, respectively. The statistical comparison revealed that there were significant differences in the primary ISQ value and percussion sound frequency between D1 and D2 and between D1 and D3 bone. No significant differences in the primary ISQ value and percussion sound frequency were found between D2 and D3.

From the corresponding results of primary ISQ and implant percussion sound frequency in each bone type, statistically significant positive correlations were found between the primary ISQ and the percussion sound frequency (*r* = 0.606; *p* < 0.001), revealing a moderate correlation between the primary ISQ and percussion sound frequency. This indicates that higher implant percussion sound frequencies have a greater primary ISQ value than lower implant percussion sound frequencies. This result corresponds with the outcome of classifying implants based on primary ISQ value, which revealed that implants with primary ISQ < 80 had a significantly lower percussion sound frequency than implants with primary ISQ 80–85 and implants with primary ISQ > 85.

In terms of the possibility that the level of percussion force influences the percussion sound frequency, it has been established that the percussion force only influences the sound intensity level measured in decibels (dB) and does not have any effect on the sound frequency. In addition, this study has percussion calibration with an intra‐rater reliability of 0.971, indicating a high degree of accuracy and precision between multiple administrations of a percussion test performed by a single examiner. In addition, percussion tests were performed in a direction parallel to the implant's long axis to address potential discrepancies in the percussion direction, which can affect the frequency of the percussion sound due to vibration patterns resulting from the quality of the surrounding bone in various directions. However, in this study, percussion tests were performed on healing abutments of pig ribs where all of the implants were similar in both diameter and length. Furthermore, the position of the implant has a relevant effect. For example, maxillary dental implants may have a higher sound frequency because of their proximity to the nasal cavity and maxillary sinus (Zanetti et al. 2018).

In addition to the influence of bone density on implant stability, the duration of the healing process also affects ISQ values. Implant stability changes with altered bone‐to‐implant contact and bone remodeling. At the time of implant placement, the ISQ value depends on the level of primary bone contact and the biomechanical properties of the bone around the implant. Subsequently, ISQ values decrease between 2 and 4 weeks after surgery (Chen et al. 2019). This decrease in ISQ value is caused by the bone relaxing after being compressed, biological changes that happen during early bone healing, and the start of bone resorption at the marginal bone crest (Glauser Lundgren and Gottlow 2003). After a stabilization period, the ISQ value started to increase again because of bone formation or remodeling (Nedir et al. 2004; Salvi and Lang 2004). Therefore, the ISQ value and percussion sound may vary at any time during the healing process. However, the percussion test can create implant micromovement, which can affect and sensitize the early stage of the osseointegration process (Brunski 1999; Zanetti et al. 2018). Therefore, it is not recommended to perform the percussion test during the first 2 to 4 weeks after implant placement or during the early stages of bone healing.

In the present experimental conditions, there is an overall positive correlation between the primary ISQ value and the percussion sound frequency. This indicates that implants with a higher percussion sound frequency generally tend to have greater primary stability than those with a lower frequency. In clinical practice, alterations in percussion sound could be used to monitor changes in implant stability, which could potentially signal concern related to the progress of osseointegration, peri‐implantitis, or bone loss. However, it is crucial to note that these findings are based on an in vitro study and may not be directly comparable to a clinical situation. Further clinical studies are necessary to validate the hypotheses proposed in this study.

## 5 Conclusions

Within the limitations of this in vitro study, it can be concluded that the primary ISQ value and the percussion sound frequency are positively correlated. Implant percussion is a practical method to assess implant stability, as implants with high‐frequency percussion sounds tend to have more primary implant stability than those with low‐frequency percussion sounds; however, further studies are needed to provide more information.

## Author Contributions

Data collection, data analysis, and drafting of the article by Wansiri Peeraprasompong. Critical revision of the article by Weerapan Aunmeungtong. Approval of article and funding secured by Pathawee Khongkhunthian.

## Conflicts of Interest

The authors declare no conflicts of interest.

## Data Availability

Data for this study are available from the corresponding author upon request.
